# 3-Factor prothrombin complex concentrate versus 4-factor prothrombin complex concentrate for the reversal of oral factor Xa inhibitors

**DOI:** 10.1007/s11239-024-03052-4

**Published:** 2024-10-28

**Authors:** William Blake Hays, Kelsey Billups, Jessica Nicholson, Abby M. Bailey, Haili Gregory, Erin R. Weeda, Kyle A. Weant

**Affiliations:** 1 Department of Pharmacy, Indiana University Health West Hospital, Avon, IN USA; 2 Department of Pharmacy Services, Medical University of South Carolina, Charleston, SC USA; 3 Department of Pharmacy, Indiana University Health Adult Academic Medical Center, Indianapolis, IN USA; 4 Department of Pharmacy Services, University of Kentucky HealthCare, Lexington, KY USA; 5 Department of Pharmacy, University of North Carolina Health, Chapel Hill, NC USA; 6 Department of Biomedical Sciences, University of South Carolina School of Medicine Greenville, Greenville, SC USA; 7 Department of Clinical Pharmacy and Outcomes Sciences, College of Pharmacy, University of South Carolina, 715 Sumter Street—CLS 316A, Columbia, SC 29208 USA

**Keywords:** Prothrombin complex concentrations, Factor Xa inhibitors, Hemostatics, Hemorrhage, Factor VII, Factor IX, Thromboembolism, Platelet aggregation inhibitors

## Abstract

Multiple agents exist for the reversal of oral Factor Xa inhibitor (FXa) associated bleeding, including Coagulation FXa Recombinant, Inactivated zhzo (andexanet alfa) and 4-factor prothrombin complex concentrate (4F-PCC). While classified as a 3F-PCC product, Profilnine contains up to 35 IU of Factor VII (per 100 IU of Factor IX) in addition to therapeutic levels of Factors II, IX, and X, and has demonstrated a similar impact on prothrombin time and blood product usage in non-warfarin related bleeding. This was a retrospective, multicenter study at four medical centers of adult patients who presented with major bleeding associated with oral FXa inhibitors and received either 4F-PCC (n = 64) or 3F-PCC (n = 61). The primary outcome was hemostatic effectiveness. Secondary outcomes included the incidence of thromboembolism, in-hospital mortality, and length of stay. The most common indication for reversal was intracranial bleeding. For the primary outcome, 84% of all patients were rated as effective with no difference noted between the groups (p = 0.81). No significant difference between groups was found in the multivariable analysis adjusting for baseline differences between groups including race, total body weight, type of bleeding, and the use of antiplatelet therapy. There was no difference in the length of stay, in-hospital mortality, or the incidence of thromboembolism between the groups. Overall, no significant differences were found in the effectiveness or safety of 4F-PCC and 3F-PCC use in the management of oral FXa inhibitor-associated bleeding. Further investigations are warranted to explore the use of 3F-PCC for this indication and its safety and effectiveness.

**Graphical Abstract:**

3-Factor Prothrombin Complex Concentrate versus 4-Factor Prothrombin Complex Concentrate for the Reversal of Oral Factor Xa Inhibitors.

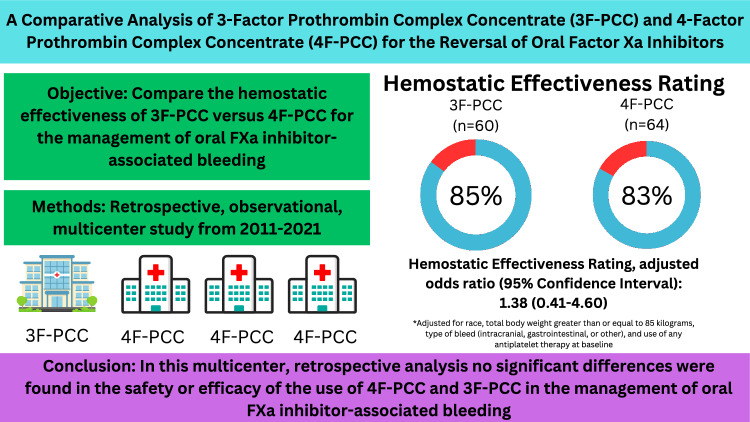

**Supplementary Information:**

The online version contains supplementary material available at 10.1007/s11239-024-03052-4.

## Highlights

- Some institutions currently use 3-factor prothrombin complex concentrate (3F-PCC) [Profilnine] for reversal of oral FXa inhibitor-associated bleeding rather than 4F-PCC because it has similar amount of Factor VII content compared to 4-factor prothrombin complex concentrate (4F-PCC) [Cofact], and an associated lower cost.
- The current study compared the effectiveness of 4F-PCC and 3F-PCC in reversing bleeding associated with oral Factor Xa inhibitors, finding no significant difference in hemostatic effectiveness between the two groups (84% effectiveness overall).
- Both 4F-PCC and 3F-PCC demonstrated similar safety profiles, with no significant difference in thromboembolism incidence, in-hospital mortality, or length of hospital stay.
- The findings suggest that 3F-PCC may be as effective and safe as 4F-PCC for the management of oral FXa inhibitor-associated bleeding, highlighting the need for further research to confirm these results and evaluate the cost-benefit of using 3F-PCC.

## Introduction

The prescribing rates of oral factor Xa (FXa) inhibitors such as rivaroxaban, apixaban, and edoxaban continue to increase throughout the United States, providing a safe and effective alternative to traditional Vitamin K antagonists [1]. Despite their documented increased safety when compared to agents such as warfarin, bleeding secondary to their use remains a concern with these agents. As the prevalence of these agents continues to increase, the identification of an optimal reversal strategy for oral FXa inhibitor-associated bleeding has become increasingly critical but controversy exists regarding available approaches. Current guidelines recommend the use of Coagulation FXa Recombinant, Inactivated zhzo (andexanet alfa) for the reversal of FXa inhibitors and the use of alternative options such as 4-factor prothrombin complex concentrate (4F-PCC) or activated PCC (aPCC) if it is not readily available [2–4]. The Neurocritical Care Society continues to only suggest the administration of 4F-PCC or aPCC for reversal of FXa inhibitors, however the publication of these guidelines in 2016 predates the Food and Drug Administration approval of Andexanet alfa in 2018 [5].

Although multiple studies exist supporting the use of 4F-PCC for reversal of FXa inhibitors, the majority of this data is considered low quality evidence [5]. Despite this, the available data does demonstrate the potential for a high rate of hemostasis (~ 80%) and a low rate of thrombosis (~ 4%) [6]. Even when compared head-to-head to andexanet alfa, patients who received 4F-PCC for intracranial hemorrhage have demonstrated similar stability of head computed tomography (CT) scans at 6 and 24 h after reversal [7]. Kcentra and Cofact, two 4F-PCC agents, contain approximately 55.1 IU and 28–80 IU of Factor VII (per 100 IU of Factor IX), respectively. Interestingly, while classified as a 3F-PCC product, Profilnine contains up to 35 IU of Factor VII (per 100 IU of Factor IX) in addition to therapeutic levels of Factors II, IX, and X [5, 8, 9]. When compared clinically, Profilnine and KCentra have demonstrated a similar impact on prothrombin time in those on rivaroxaban and similar blood product usage in non-warfarin related bleeding [10, 11]. It also should be noted that Cofact was the 4F-PCC product first studied in reversal of rivaroxaban [12]. Having a similar amount of Factor VII compared to 4F-PCC Cofact, and an associated lower cost, some institutions currently use 3F-PCC Profilnine for reversal of oral FXa inhibitor-associated bleeding rather than 4F-PCC. Although these agents have been used for some time, there is currently a paucity of literature comparing 3F-PCC to 4F-PCC for the management of oral FXa inhibitor-associated bleeding. Given this gap in the literature, the purpose of this study is to compare the hemostatic effectiveness and safety of 3F-PCC (Profilnine) versus 4F-PCC (KCentra) for the management of oral FXa inhibitor-associated bleeding.

## Methods

This was a retrospective, observational, multicenter study that identified patients treated at the Medical University of South Carolina, University of Kentucky HealthCare, University of Florida Health, and Indiana University Health Methodist Hospital between 2011 and 2021. The study was approved by the institutional review board at all study sites, with the data coordinating site being the University of South Carolina. Adult patients over 18 years of age who presented for major bleeding associated with oral factor Xa inhibitors were identified for inclusion. Major bleeding was defined as bleeding into a critical site, life-threatening bleeding requiring emergent surgery or an invasive procedure, or bleeding requiring a blood transfusion within 24 h of reversal [13]. Patients had to receive either 4F-PCC or 3F-PCC in order to be included. As 3F-PCC was used as the standard product at Indiana University Health Methodist Hospital during the study period, all patients receiving 3F-PCC were identified from this center, while patients receiving 4F-PCC identified from the remaining 3 centers. Exclusion criteria included pregnancy, incarceration, perioperative reversal unrelated to bleeding, intracranial hemorrhage (ICH) with an initial Glasgow Coma Scale (GCS) less than seven, death within 24 h of reversal, acute coronary syndrome or ischemic stroke within the last 30 days, initial presentation at outside hospital, surgery within 24 h of presentation, large blood vessel rupture (e.g. aortic dissection or ruptured aortic aneurysm), primary intraventricular hemorrhage, epidural hematoma, or if the patient underwent a craniotomy prior to repeat head imaging.

Data collection was performed by manual chart review of electronic medical records. Study data were collected and managed using REDCap (Research Electronic Data Capture). REDCap access was supported by the South Carolina Clinical & Translational Research Institute, with an academic home at the Medical University of South Carolina, through NIH—NCATS Grant Number UL1 TR001450 [14]. Interrater reliability (IRR) testing was conducted at all sites to ensure consistency with an IRR of 1. Baseline data collected included demographics, oral factor Xa inhibitor utilized, indication for the oral factor Xa inhibitor, use of antiplatelet agents, and type of bleeding noted. Laboratory parameters collected included markers of blood volume (e.g. hemoglobin, hematocrit), renal function (e.g. serum creatinine) as it can impact the clearance of certain anticoagulants, and coagulation (e.g. prothrombin time, activated partial thromboplastin time). Data on reversal strategies and blood products utilized were also collected. Patients were divided into cohorts based on the use of 4F-PCC and 3F-PCC. The primary outcome of interest was hemostatic effectiveness, which was assessed via previously validated criteria [13]. Secondary outcomes included thromboembolism, in-hospital mortality, and length of hospital and intensive care unit stay.

Data are presented as counts with percentage for categorical data and medians with interquartile ranges for continuous data. Chi square or Fisher’s Exact tests were used to compare categorical variables between cohorts, while Mann–Whitney U tests were utilized for continuous variables. To assess the primary outcome of hemostatic effectiveness in patients who received 4F-PCC versus 3F-PCC after adjusting for baseline factors, adjusted odds ratios and 95% confidence intervals were estimated using logistic regression. This multivariable analysis adjusted for race, total body weight greater than or equal to 85 kg, type of bleed (intracranial, gastrointestinal, or other), and use of any antiplatelet therapy at baseline. Statistical analyses were conducted using SPSS v29 (IBM Corp., Armonk, NY).

## Results

**Fig. 1:**
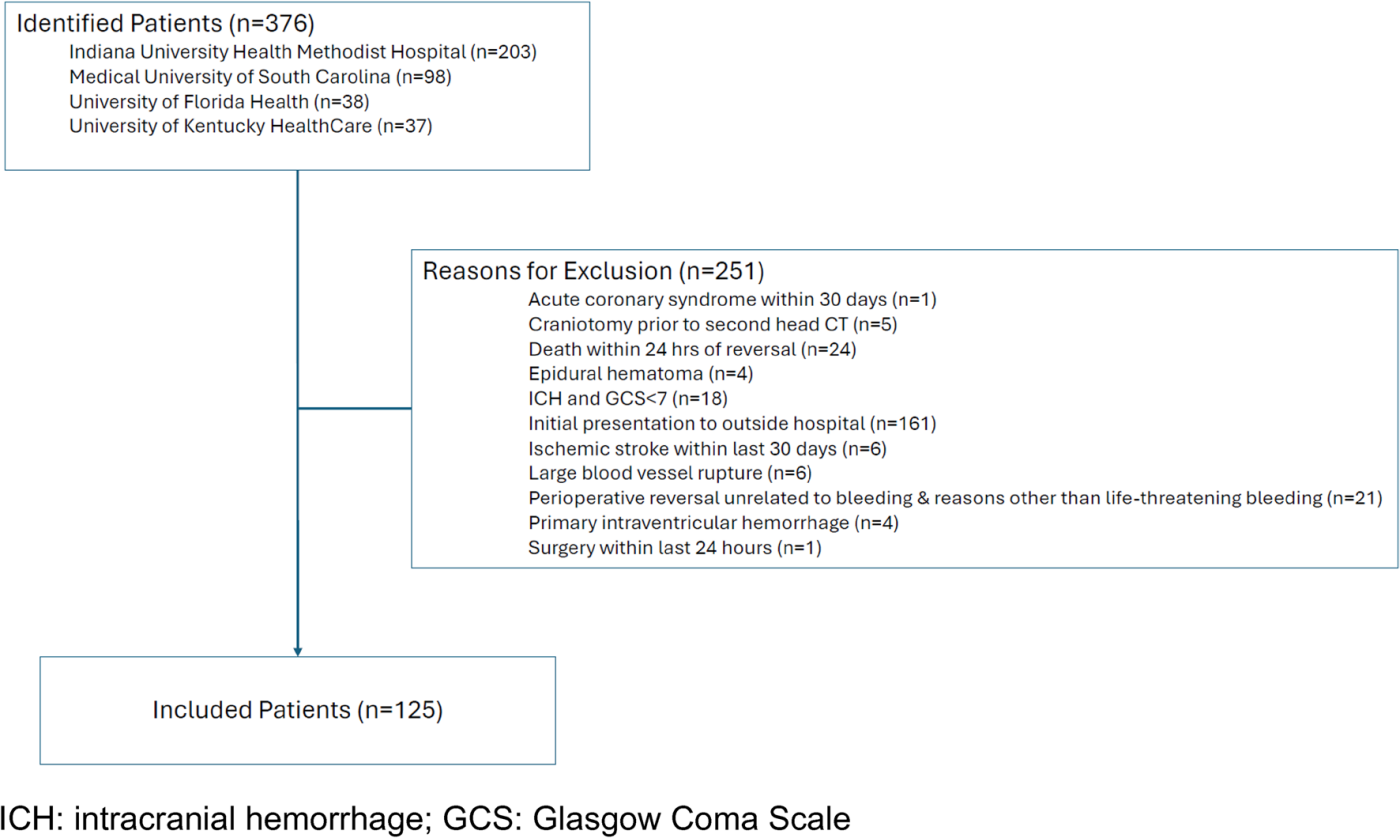
Schematic of patient selection process

Of the 376 patients screened for inclusion across the four centers, 251 did not meet criteria for inclusion (Fig. 1). The most common reason for exclusion was initial presentation to an outside hospital (n = 161). Of the remaining 125 that were included in our analysis, 64 received 4F-PCC and 61 received 3F-PCC for the management of oral FXa inhibitor-associated bleeding (Table 1). The median age of included patients was 75 years (interquartile range = 64–81 years) and the most common indication for reversal was ICH. Apixaban was the most common FXa inhibitor used at baseline. Table 2 includes data on reversal strategies and blood product utilization. The median dose of PCC product was just under 50 units per kilogram of total body weight in both groups. For the primary outcome of hemostatic effectiveness, 84% of all patients were rated as effective, of which 81.1% was rated as “excellent” and 18.9% was rated as “good” (Table 3). There was no difference in this primary outcome between 4F-PCC and 3F-PCC groups (p = 0.81). The adjusted analysis also did not identify any significant difference. Secondary outcomes are included in supplementary file 1. Thromboembolism occurred in 7 patients, with no difference between 4F-PCC and 3F-PCC groups. There was also no difference in length of stay or in-hospital mortality between groups.

**Table 1:** Baseline demographics and clinical characteristics

Baseline characteristics	4F-PCC (n = 64)	3F-PCC (n = 61)	p-value
Age, years, median (IQR)	76 (15.5)	74 (17.0)	0.151
Total Body Weight, kg, median (IQR)	74.7 (28.9)	86.0 (43.4)	0.089
Total Body Weight ≥ 85 kg, n (%)	22 (34.3)	32 (52.5)	0.041
Height, inches, median (IQR)	67.0 (6.9)	67.0 (7.0)	0.576
Ideal Body Weight, kg, median (IQR)	62.70 (19.2)	63.8 (20.6)	0.632
Adjusted Body Weight, kg, median (IQR)	66.69 (19.2)	72.3 (29.0)	0.171
Body Mass Index, kg/m^2^, median (IQR)	26.73 (7.4)	29.7 (10.9)	0.099
Male, n (%)	30 (46.9)	30 (49.2)	0.797
Race, n (%)			
White/Caucasian	57 (89.1)	42 (68.9)	0.011
Black/African-American	7 (10.9)	17 (27.9)	
Other	0 (0)	2 (3.3)	
Indication for anticoagulation, n (%)			
Atrial fibrillation	47 (73.4)	37 (60.7)	0.189
Venous thromboembolism	16 (25.0)	20 (32.8)	
Other	1 (1.6)	4 (6.6)	
Patients on apixaban, n (%)	45 (70.3)	44 (72.1)	0.846
Daily dose, mg, median (IQR)	10 (0)	10 (0)	0.468
Patients on rivaroxaban, n (%)	17 (26.6)	17 (27.9)	0.846
Daily dose, mg, median (IQR)	20 (5)	20 (0)	0.514
Patients on edoxaban, n (%)	2 (3.1)	0 (0)	0.975
Daily dose, mg, median (IQR)	60 (0)	–	
Antiplatelet Use, n (%) None Aspirin only Clopidogrel/ticagrelor/prasugrel only Aspirin + clopidogrel/ticagrelor/prasugrel	16 (25.0) 48(75.0) 12(18.8) 2 (3.1) 2(3.1)	24 (39.3) 37 (60.7) 24 (39.3) 0 (0) 0 (0)	0.124
Laboratory Parameters, median (IQ R) Hemoglobin Hematocrit Creatinine Creatinine Clearance (mL/min) Prothrombin Time (sec) International normalized ratio Activated partial thromboplastin time (sec)	11.9 (2.7) 37.0 (8.5) 1.0 (0.5) 58.5 (46.1) 16.4 (3.6) 1.3 (0.4) 31.4 (7.2)	10.4 (5.9) 32.7 (17.2) 1.2 (0.9) 56.7 (54.5) 17.6 (9.9) 1.6 (0.8) 32.1 (10.0)	0.059 0.031 0.008 0.802 0.035 < 0.001 0.594
Creatinine clearance, n (%)			
< 30 mL/min	5 (7.8)	11 (18.0)	0.205
30–60 mL/min	30 (46.9)	23 (37.7)	
> 60mL/min	29 (45.3)	27 (44.3)	
Type of bleed, n (%)			
Intracranial hemorrhage	50 (78.1)	20 (32.7)	< 0.001
Intracerebral	15 (23.4)	10 (16.4)	
Subdural	14 (21.9)	10 (16.4)	
Subarachnoid	3 (4.7)	5 (8.2)	
Traumatic in origin	22 (34.4)	7 (11.5)	
Pericardial	1 (1.6)	1 (1.6)	> 0.999
Musculoskeletal	2 (3.1)	5 (8.2)	0.266
Intra-spinal	0 (0)	1 (1.6)	0.488
Intrathoracic	1 (1.6)	1 (1.6)	> 0.999
Gastrointestinal	3 (4.7)	24 (39.3)	< 0.001
Epistaxis	0 (0)	1 (1.6)	0.488
Other	7 (10.9)	8 (13.1)	0.787
Invasive interventions, n (%)			
None	40 (62.5)	43 (70.5)	0.449
Sternotomy	0 (0)	0 (0)	> 0.999
Evacuation of intracranial hematoma	4 (6.3)	2 (3.3)	0.680
Pericardiocentesis	1 (1.6)	1 (1.6)	> 0.999
Spinal decompression	0 (0)	0 (0)	> 0.999
Endoscopy with cauterization	1 (1.6)	4 (6.6)	0.200
External Ventricular Drain Placement	4 (6.3)	2 (3.3)	0.680
Interventional Radiology	3 (4.7)	1 (1.6)	0.619
Other	4 (6.3)	4 (6.6)	> 0.999

*4F-PCC* 4-factor prothrombin complex concentrate, *3F-PCC* 3-factor prothrombin complex concentrate, *IQR* Interquartile range

**Table 2:** Reversal agents and blood products

	4F-PCC (n = 64)	3F-PCC (n = 61)	p-value
Dose, units, median (IQR)	3399 (1567)	3500 (1960)	0.999
Dose, units/kg total BW, median (IQR)	47.8 (9.6)	46.5 (14.2)	0.153
Dose, units/kg ideal BW, median (IQR)	56.5 (20.6)	55.0 (24.5)	0.875
Dose, units/kg adjusted BW, median (IQR)	52.8 (12.5)	50.7 (18.3)	0.462
Time from ED admission to PCC administration, hrs, median (IQR)	2 (2.8)	3 (2)	0.307
Repeat dose, n (%)	2 (3.1)	0 (0)	0.496
Blood products, n (%)			
Packed red blood cells	12 (18.8)	28 (45.9)	0.002
Platelets	5 (7.8)	5 (8.2)	> 0.999
Cryoprecipitate	0 (0)	0 (0)	> 0.999
Fresh frozen plasma	3 (4.7)	6 (9.8)	0.316
Whole blood	0 (0)	0 (0)	> 0.999
Blood products, units, median (IQR)			
Packed red blood cells	2 (3.5)	3 (2)	0.468
Platelets	1 (0.5)	6 (6)	0.008
Cryoprecipitate	0 (0)	0 (0)	> 0.999
Fresh frozen plasma	2 (1)	2.5 (5)	0.357
Whole blood	0 (0)	0 (0)	> 0.999

*4F-PCC* 4-factor prothrombin complex concentrate, *3F-PCC* 3-factor prothrombin complex concentrate, *IQR* Interquartile range, *BW* Body weight, *ED* Emergency Department

**Table 3:** Primary outcome

	4F-PCC (n = 64)	3F-PCC* (n = 60)	p-value
Hemostatic effectiveness rating, n (%)			
Effective	53 (82.8)	51 (85.0)	0.81
Hemostatic effectiveness rating, adjusted odds ratio (95% confidence interval)**			
Adjusted	1.38 (0.41–4.60)		

^*^One patient was transferred to hospice and so outcome unable to be assessed

^**^4F-PCC served as the reference group. Adjusted for race, total body weight greater than or equal to 85 kg, type of bleed (intracranial, gastrointestinal, or other), and use of any antiplatelet therapy at baseline

*4F-PCC* 4-factor prothrombin complex concentrate, *3F-PCC* 3-factor prothrombin complex concentrate

## Discussion

In this retrospective analysis of 125 patients across four medical centers, no significant differences were found in the safety or efficacy of the use of 4F-PCC and 3F-PCC in the management of oral FXa inhibitor-associated bleeding. Additionally, in the multivariable analysis adjusting for baseline differences between groups including race, total body weight greater than or equal to 85 kg, type of bleed (intracranial, gastrointestinal, or other), and use of any antiplatelet therapy at baseline, no significant differences were noted.

Copious amounts of data exist for the use of PCC in the treatment of bleeding secondary to warfarin therapy and multiple studies have sought to compare the use of 3F-PCC to 4F-PCC for this indication with mixed results [15]. On the other hand, existing data comparing the utilization of 3F-PCC and 4F-PCC in the reversal of oral FXa anticoagulation is quite limited. One open-label, single-center study compared the effects of the administration of 3F-PCC with 4F-PCC on the pharmacodynamics of rivaroxaban in 35 healthy volunteers after receiving four days of therapy [11]. Participants received a 50 IU/kg bolus dose of either 4F-PCC, 3F-PCC, or 0.9% sodium chloride four hours after the morning dose. Within 30 min, 4F-PCC reduced the mean prothrombin time by 2.5–3.5 s, depending on the reagent, whereas 3F-PCC produced only a 0.6–1.0 s reduction. However, 3F-PCC was found to reverse rivaroxaban-induced changes in thrombin generation faster than 4F-PCC. This study demonstrates the potential of both 3F-PCC and 4F-PCC to at least partially reverse the anticoagulant effects of rivaroxaban in healthy adults. The disparity in the results between the two agents could be theorized to be secondary to the concentrations of individual factors contained within each product. To the authors’ knowledge, the only other clinical study to compare the use of 3F-PCC and 4F-PCC in the treatment of bleeding secondary to FXa inhibitor administration was a study that looked at patients treated for bleeding due to non-warfarin-related indications at two hospitals during a 19-month period [10]. Of the 182 patients included in the study, only 5 (2.7%) received either PCC for reversal of FXa-associated bleeding. Nevertheless, the proportion of patients receiving fresh frozen plasma (FFP) and the median volumes of plasma received did not significantly differ between the two groups and both groups had similar blood product use, intensive care unit (ICU) length of stay (LOS), and in-hospital mortality. Our investigation greatly expanded upon this data by evaluating a diverse population with FXa associated bleeding and comparing the two PCC agents in a multicenter fashion.

Our study is strengthened by the use of a standard definition for major bleeding as well as the use of a validated measure to assess hemostatic effectiveness. The latter is notable as some retrospective studies of PCC have relied on more subjective measures, such as clinical judgement, to assess hemostatic effectiveness [16]. Our study is limited by its retrospective nature and reliance on accurate documentation in electronic health record. However, the accurate documentation of PCC and blood product usage is one of particular focus at most facilities due to cost and safety reasons, hence it is less likely to be omitted or incorrectly documented. There was also variation noted between the groups with regards to baseline characteristics, including bleeding site, which is to be expected by the nature of a multicenter study. We attempted to mitigate the impact of these variances to the degree possible through the use of multivariable analysis which demonstrated no significant differences between the two groups. Lastly, we excluded ~ 160 individuals because they presented to outside hospitals prior to their encounter at the included centers. Although this decreases the applicability of our findings, it increases the internal validity by ensuring that PCC and blood product usage was accurately captured and not omitted because we could not review records from the outside institutions.

## Conclusion

In this retrospective, multicenter analysis comparing the use of 4F-PCC and 3F-PCC in the reversal of oral FXa associated bleeding, no significant differences were found in the effectiveness of the two agents. There was also no difference found in incidence of thromboembolism, the length of hospital stay, or in-hospital mortality between groups. Further investigations are warranted to explore the use of 3F-PCC for this indication and its comparative safety and effectiveness.

## Supplementary Information

Below is the link to the electronic supplementary material.

Supplementary file1 (MP4 31 KB)

## References

[1] Kattoor AJ, Pothineni NV, Goel A, Syed M, Syed S, Paydak H et al (2019) Prescription patterns and outcomes of patients with atrial fibrillation treated with direct oral anticoagulants and warfarin: a real-world analysis. J Cardiovasc Pharmacol Ther 24(5):428–434. 10.1177/1074248419841634 31035795 10.1177/1074248419841634

[2] Christensen H, Cordonnier C, Korv J, Lal A, Ovesen C, Purrucker JC et al (2019) European stroke organisation guideline on reversal of oral anticoagulants in acute intracerebral haemorrhage. Eur Stroke J 4(4):294–306. 10.1177/2396987319849763 31903428 10.1177/2396987319849763 PMC6921939

[3] Tomaselli GF, Mahaffey KW, Cuker A, Dobesh PP, Doherty JU, Eikelboom JW et al (2020) 2020 ACC expert consensus decision pathway on management of bleeding in patients on oral anticoagulants: a report of the American college of cardiology solution set oversight committee. J Am Coll Cardiol 76(5):594–622. 10.1016/j.jacc.2020.04.053 32680646 10.1016/j.jacc.2020.04.053

[4] Baugh CW, Levine M, Cornutt D, Wilson JW, Kwun R, Mahan CE et al (2020) Anticoagulant reversal strategies in the emergency department setting: recommendations of a multidisciplinary expert panel. Ann Emerg Med 76(4):470–485. 10.1016/j.annemergmed.2019.09.001 31732375 10.1016/j.annemergmed.2019.09.001 PMC7393606

[5] Frontera JA, Lewin JJ 3rd, Rabinstein AA, Aisiku IP, Alexandrov AW, Cook AM et al (2016) Guideline for reversal of antithrombotics in intracranial hemorrhage: a statement for healthcare professionals from the neurocritical care society and society of critical care medicine. Neurocrit Care 24(1):6–46. 10.1007/s12028-015-0222-x 26714677 10.1007/s12028-015-0222-x

[6] Panos, N. G., Cook, A. M., John, S., Jones, G. M., & Neurocritical Care Society Pharmacy Study, G (2020) Factor Xa inhibitor-related intracranial hemorrhage: results from a multicenter, observational cohort receiving prothrombin complex concentrates. Circulation 141(21):1681–1689. 10.1161/CIRCULATIONAHA.120.045769 32264698 10.1161/CIRCULATIONAHA.120.045769

[7] Ammar AA, Ammar MA, Owusu KA, Brown SC, Kaddouh F, Elsamadicy AA et al (2021) Andexanet alfa versus 4-factor prothrombin complex concentrate for reversal of factor Xa inhibitors in intracranial hemorrhage. Neurocrit Care 35(1):255–261. 10.1007/s12028-020-01161-5 33403588 10.1007/s12028-020-01161-5 PMC10273779

[8] CSL Behring. Kcentra (Prothrombin Complex Concentrate (Human)) [package insert]. U.S. Food and Drug Administration. website. https://www.fda.gov/media/85512/download?attachment. Accessed May 10, 2024.

[9] Grifols Biologicals Inc. Profilnine SD (Factor IX Complex) [package insert]. U.S. Food and Drug Administration. website. https://www.fda.gov/media/80956/download. Accessed May 10, 2024.

[10] DeAngelo J, Jarrell DH, Cosgrove R, Camamo J, Edwards CJ, Patanwala AE (2018) Comparison of blood product use and costs with use of 3-factor versus 4-factor prothrombin complex concentrate for off-label indications. Am J Health Syst Pharm 75(15):1103–1109. 10.2146/ajhp180076 29941507 10.2146/ajhp180076

[11] Levi M, Moore KT, Castillejos CF, Kubitza D, Berkowitz SD, Goldhaber SZ et al (2014) Comparison of three-factor and four-factor prothrombin complex concentrates regarding reversal of the anticoagulant effects of rivaroxaban in healthy volunteers. J Thromb Haemost 12(9):1428–1436. 10.1111/jth.12599 24811969 10.1111/jth.12599

[12] Eerenberg ES, Kamphuisen PW, Sijpkens MK, Meijers JC, Buller HR, Levi M (2011) Reversal of rivaroxaban and dabigatran by prothrombin complex concentrate: a randomized, placebo-controlled, crossover study in healthy subjects. Circulation 124(14):1573–1579. 10.1161/CIRCULATIONAHA.111.029017 21900088 10.1161/CIRCULATIONAHA.111.029017

[13] Sarode R, Milling TJ Jr, Refaai MA, Mangione A, Schneider A, Durn BL et al (2013) Efficacy and safety of a 4-factor prothrombin complex concentrate in patients on vitamin K antagonists presenting with major bleeding: a randomized, plasma-controlled, phase IIIb study. Circulation 128(11):1234–1243. 10.1161/CIRCULATIONAHA.113.002283 23935011 10.1161/CIRCULATIONAHA.113.002283 PMC6701181

[14] Harris PA, Taylor R, Thielke R, Payne J, Gonzalez N, Conde JG (2009) Research electronic data capture (REDCap)–a metadata-driven methodology and workflow process for providing translational research informatics support. J Biomed Inform 42(2):377–381. 10.1016/j.jbi.2008.08.010 18929686 10.1016/j.jbi.2008.08.010 PMC2700030

[15] Voils SA, Baird B (2012) Systematic review: 3-factor versus 4-factor prothrombin complex concentrate for warfarin reversal: does it matter? Thromb Res 130(6):833–840. 10.1016/j.thromres.2012.10.001 23137921 10.1016/j.thromres.2012.10.001

[16] Costa OS, Baker WL, Roman-Morillo Y, McNeil-Posey K, Lovelace B, White CM et al (2020) Quality evaluation of case series describing four-factor prothrombin complex concentrate in oral factor Xa inhibitor-associated bleeding: a systematic review. BMJ Open 10(11):e040499. 10.1136/bmjopen-2020-040499 33154059 10.1136/bmjopen-2020-040499 PMC7646359

