# Supplementary material for: 3-Factor prothrombin complex concentrate versus 4-factor prothrombin complex concentrate for the reversal of oral factor Xa inhibitors
Source: J Thromb Thrombolysis. 2024 Oct 28;58(2):276–83. doi: 10.1007/s11239-024-03052-4 (PMC11885330; doi:10.1007/s11239-024-03052-4)
Supplement: Supplementary file 1 — Supplementary file1 (MP4 31 KB) [file 11239_2024_3052_MOESM1_ESM.docx]

**eTable 1. Additional patient outcomes**

|  | 4F-PCC  (n=64) | 3F-PCC*  (n=60) | p-value |
| --- | --- | --- | --- |
| Location of bleed with ineffective hemostasis, n (%) |  |  |  |
| Pericardial tamponade | 0 (0) | 0 (0) | >0.999 |
| Intracerebral | 3 (4.7) | 2 (3.3) | >0.999 |
| Subdural | 1 (1.6) | 1 (1.6) | >0.999 |
| Subarachnoid | 2 (3.1) | 2 (3.3) | >0.999 |
| Traumatic Intracranial | 6 (9.4) | 0 (0) | 0.028 |
| Musculoskeletal | 0 (0) | 0 (0) | >0.999 |
| Intra-Spinal | 0 (0) | 0 (0) | >0.999 |
| Intrathoracic | 0 (0) | 0 (0) | >0.999 |
| Gastrointestinal | 1 (1.6) | 5 (8.2) | 0.106 |
| Epistaxis | 0 (0) | 0 (0) | >0.999 |
| Other | 3 (4.7) | 1 (1.6) | 0.620 |
| Thromboembolic events, n (%) | 3 (4.7) | 4 (6.6) | 0.711 |
| Length of ICU stay, day, median (IQR) | 3.0 (4.8) | 3.0 (6.0) | 0.352 |
| Length of hospital stay, day, median (IQR) | 7.5 (10.0) | 8.0 (7.0) | 0.623 |
| In-hospital mortality, n (%) | 9 (14.1) | 9 (14.8) | >0.999 |

*One patient was transferred to hospice and so outcome was unable to be assessed

4F-PCC: 4-factor prothrombin complex concentrate; 3F-PCC: 3-factor prothrombin complex concentrate; ICU: Intensive Care Unit; IQR: Interquartile Range
